# Leg and Joint Stiffness in Children with Spastic Diplegic Cerebral Palsy during Level Walking

**DOI:** 10.1371/journal.pone.0143967

**Published:** 2015-12-02

**Authors:** Ting-Ming Wang, Hsing-Po Huang, Jia-Da Li, Shih-Wun Hong, Wei-Ching Lo, Tung-Wu Lu

**Affiliations:** 1 Department of Orthopaedic Surgery, School of Medicine, National Taiwan University, Taipei, Taiwan, R.O.C; 2 Institute of Biomedical Engineering, National Taiwan University, Taipei, Taiwan, R.O.C; Rutgers University -New Jersey Medical School, UNITED STATES

## Abstract

Individual joint deviations are often identified in the analysis of cerebral palsy (CP) gait. However, knowledge is limited as to how these deviations affect the control of the locomotor system as a whole when striving to meet the demands of walking. The current study aimed to bridge the gap by describing the control of the locomotor system in children with diplegic CP in terms of their leg stiffness, both skeletal and muscular components, and associated joint stiffness during gait. Twelve children with spastic diplegia CP and 12 healthy controls walked at a self-selected pace in a gait laboratory while their kinematic and forceplate data were measured and analyzed during loading response, mid-stance, terminal stance and pre-swing. For calculating the leg stiffness, each of the lower limbs was modeled as a non-linear spring, connecting the hip joint center and the corresponding center of pressure, with varying stiffness that was calculated as the slope (gradient) of the axial force vs. the deformation curve. The leg stiffness was further decomposed into skeletal and muscular components considering the alignment of the lower limb. The ankle, knee and hip of the limb were modeled as revolute joints with torsional springs whose stiffness was calculated as the slope of the moment vs. the angle curve of the joint. Independent t-tests were performed for between-group comparisons of all the variables. The CP group significantly decreased the leg stiffness but increased the joint stiffness during stance phase, except during terminal stance where the leg stiffness was increased. They appeared to rely more on muscular contributions to achieve the required leg stiffness, increasing the muscular demands in maintaining the body posture against collapse. Leg stiffness plays a critical role in modulating the kinematics and kinetics of the locomotor system during gait in the diplegic CP.

## Introduction

Cerebral palsy (CP) is an irreversible and non-progressive disorder caused by brain injury before, during or shortly after birth [1]. During growth, impaired motor control often develops with secondary abnormalities such as bony deformities and changes in the joint and muscle properties [2], leading to deviations in postures and movements. Depending on the level of involvement, children with CP may have different gait deviations, including increased hip flexion, increased knee flexion, decreased ankle dorsiflexion [1, 3], increased knee extensor moments [4], and increased hip extensor moments [5] during stance phase. Treatments may also have different effects on these gait deviations. Therefore, interpreting the measured gait deviations and their changes in response to treatment can be very challenging [6]. A measure for assessing the control of the lower limb as a whole during gait would be very helpful in the assessment of the effects of the pathology and the efficacy of the treatment in a clinical setting.

Human walking is achieved by a highly complex interaction of the force-bearing structures of the musculoskeletal system. While sophisticated models of the system have been used to provide insights into the gait mechanics in children with CP [7], a simplified phenomenological gait model can be useful for providing an overall measure of the control of gait in clinical applications. The mass-spring model simplifies the body as a point mass supported by the leg modeled as a linear spring [8]. When a human walks, the leg spring is compressed and uncompressed alternately with the motion of the body mass during the stance phase, storing and returning the elastic energy, respectively. Different stiffness of the leg spring, controlled by the muscle forces and the skeletal alignment (or joint angles), will lead to different ground reaction forces (GRF) needed to facilitate walking. Therefore, quantifying the leg stiffness, defined as the ratio of the GRF and the shortening of the lower extremity [9], for each of the lower limbs may help describe the control of the overall musculoskeletal system of the lower limb during walking and other ground contact activities [8, 10, 11].

Leg stiffness incorporates information of both the kinematics and kinetics of the lower limb joints because both the leg length and the GRF are affected by the joint angles and moments at any instance of gait [8, 9, 12]. In order to support the body against collapse during gait, both muscle torques (i.e., net internal moments) at joints, and forces transmitted in the bones and joints are needed to maintain the posture and movement while subject to the GRF, and at the same time to provide the leg stiffness required to respond to postural perturbations (i.e., changes of the length of the leg spring). Therefore, the leg stiffness can be further decomposed into the so-called skeletal and muscular components [13]. The skeletal component is related to the forces transmitted through the joints and bones, while the muscular component is closely related to the joint stiffness provided predominately by the muscular torques at the joints [10, 13] [14, 15]. According to DeVita *et al*. (2000) [13], the leg stiffness of a completely extended lower limb is provided solely by the skeletal component, and as the alignment of the limb changes to a more and more flexed posture, the contribution of the muscular component to the leg stiffness increases while that of the skeletal component decreases. An appropriate combination of both the skeletal and muscular components is necessary for meeting the requirements of anti-collapse of the limb during gait, the ratio of which will depend on the alignment of bones and the muscle moments required to maintain the posture and movement [13]. Study of the interactions between leg stiffness and joint stiffness, as well as the contributions of skeletal and muscular components, in children with CP during level walking may help explain how these children modulate their lower limb stiffness during gait in the presence of motor abnormality and other symptoms. Davis and De Luca [16] first described the ankle joint stiffness, defined as the slope of the joint angle-moment plot, and used it as an index to evaluate treatment effects in four children with CP during gait [16]. Applications have also been found in other patient groups such as children with Down syndrome [17]. However, no study has described the interactions between leg stiffness (both skeletal and muscular components) and joint stiffness of the lower limb in children with CP during walking.

The purpose of this study was to investigate the leg and joint stiffness, the contributions of skeletal and muscular components, and the associated joint kinematics and kinetics in children with spastic diplegia CP during level walking. It was hoped that the results will provide baseline data on the stiffness control of the lower limb as a whole in healthy children and in children with diplegic CP, which would be helpful in future assessment of the effects of the pathology and the efficacy of relevant treatment methods.

## Materials and Methods

### Ethics Statement

This study was approved by the Institutional Research Board of National Taiwan University Hospital (Permit Number: 201305084RINC). All the subjects and their parents/guardians were informed of the procedure and provided written informed consent prior to the study, including enrolment and data collection.

### Subjects

Twelve children with spastic diplegia cerebral palsy (age: 12.5 ± 5.2 years; height: 142.2 ± 15.9 cm; mass: 38.0 ± 12.8 kg), and twelve age-matched healthy controls (age: 11.2 ± 2.3 years; height: 141.1 ± 12.6 cm; mass: 34.4 ± 9.3 kg) participated in this study. The CP group was graded 1–3 in the Gross Motor Function Classification System (GMFCS) with slightly limited passive range of motion (ROM) of the hip and ankle, moderate to normal muscle strength and mild to normal muscle tone (Table 1). However, they were free of pain and did not show noticeable leg length discrepancy, serious muscle contracture, joint deformity or other pathology which might affect gait and/or cognitive function. The healthy controls were free from any musculoskeletal, neurological or cardiovascular disorders. An *a priori* power analysis based on pilot results on leg and joint stiffness using GPOWER [18] determined that 11 subjects per group would yield a power of 0.8 and an effect size of more than 1.15 at a significance level of 0.05.

**Table 1:** Means (standard deviations) of the ranges of motion, muscle strength, and muscle tone of the hip, knee and ankle joints for the CP group.

	Range of Motion (degree)	Muscle Strength (MMT)	Muscle Tone (Modified Ashworth Scale)
Hip			
Extension/Extensors	23.46 (7.54)	3.29 (0.60)	0.08 (0.29)
Flexion/Flexors	119.23 (2.54)	3.85 (0.61)	0.00 (0.00)
Knee			
Extension/Extensors	-0.63 (1.55)	4.02 (0.53)	0.08 (0.19)
Flexion/Flexors	134.42 (2.02)	3.75 (0.68)	0.15 (0.38)
Ankle			
Plantar-Flexion/Flexors	50.17 (3.38)	3.81 (0.49)	0.83 (0.62)
Dorsi-Flexion/Flexors	15.13 (6.39)	3.75 (0.56)	0.13 (0.31)

### Experimental protocol

Each subject walked at a self-selected pace on an 8-meter walkway. Each subject wore 39 retroreflective markers for tracking the motions of the body segments [19] using a motion analysis system (Vicon 512, OMG, UK) while the ground reaction forces (GRF) were measured using two forceplates (AMTI, USA) [20]. Before the tests, subjects were allowed to walk on the walkway several times to familiarize themselves with the experimental environment. Six successful trials were then obtained for subsequent analysis.

### Data analysis

With the measured GRF and kinematic data, angular motions and internal moments at the lower limb joints were calculated using inverse dynamics analysis [21]. Each body segment was embedded with an orthogonal coordinate system with the positive x-axis directed anteriorly, the positive y-axis superiorly and the positive z-axis to the right. A Cardanic rotation sequence (z-x-y) was used to describe the rotational movements of each joint [22, 23]. Inertial properties for each body segment were obtained using an optimization-based method [24]. A global optimization method was used to reduce the effects of soft tissue artefacts associated with the skin markers [25]. All the calculated joint moments were normalized to body weight (BW) and leg length (LL), the latter defined as the length between the anterior superior iliac spine and the medial malleolus.

**Fig 1:**
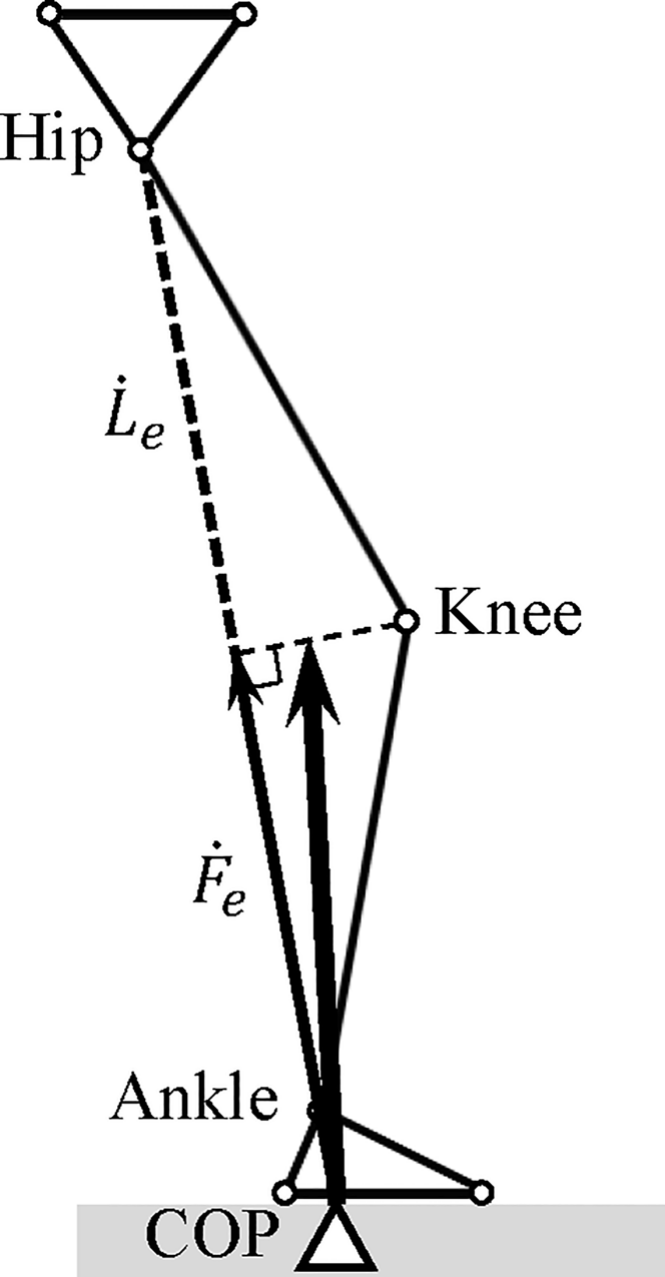
Effective GRF and effective leg length. Stick figure of a lower limb during stance phase of gait showing the definitions of the effective GRF (thin vector, Fe(t)) and effective leg length (*L*_*e*_(t)). The former is defined as the component of the measured GRF (thick vector) along the line joining the center of pressure (COP) and the hip joint center. The latter is defined as the distance between the COP and the hip joint center.

For calculating the leg stiffness, each of the lower limb was modeled as a non-linear spring, connecting the COP of the GRF of the limb and the hip joint center, with varying stiffness that could be calculated as the slope (gradient) of the force vs. deformation curve. The force applied to the leg spring, called effective GRF (*F*_*e*_(*t*)), is defined as the component of the GRF of the limb along the line joining the COP and the hip joint center. The length of the leg spring is defined as the distance between the hip joint center and the COP, and is called the effective leg length (*L*_*e*_(*t*)). Therefore, the leg stiffness, *K*_*l*_(*t*), at time *t* could be calculated as the gradient of the *F*_*e*_(*t*) vs. *L*_*e*_(*t*) curve as follows (Fig 1):

$$K_{l}\left(t\right)\;=\;\frac{{dF}_{e}\left(t\right)}{{dL}_{e}\left(t\right)}\;=\;\frac{{\dot{F}}_{e}\left(t\right)}{{\dot{L}}_{e}\left(t\right)}$$ (1)

$${\dot{F}}_{e}\left(t\right)\;=\;\frac{{dF}_{e}\left(t\right)}{dt}$$ (2)

$${\dot{L}}_{e}\left(t\right)\;=\;\frac{{dL}_{e}\left(t\right)}{dt}$$ (3)

Similarly, the joint stiffness of the ankle (*K*_*a*_(*t*)), knee (*K*_*k*_(*t*)), and hip (*K*_*h*_(*t*)) at time *t* were calculated as the slope of the moment (*M*(*t*)) vs. angle (*θ*(*t*)) curve of the joint as follows:

$$K_{j}\left(t\right)\;=\;\frac{dM\left(t\right)}{d\theta \left(t\right)}\;=\;\frac{\dot{M}\left(t\right)}{\dot{\theta }\left(t\right)}$$ (4)

$$\dot{M}\left(t\right)\;=\;\frac{dM\left(t\right)}{dt}$$ (5)

$$\dot{\theta }\left(t\right)\;=\;\frac{d\theta \left(t\right)}{dt}$$ (6)

**Fig 2:**
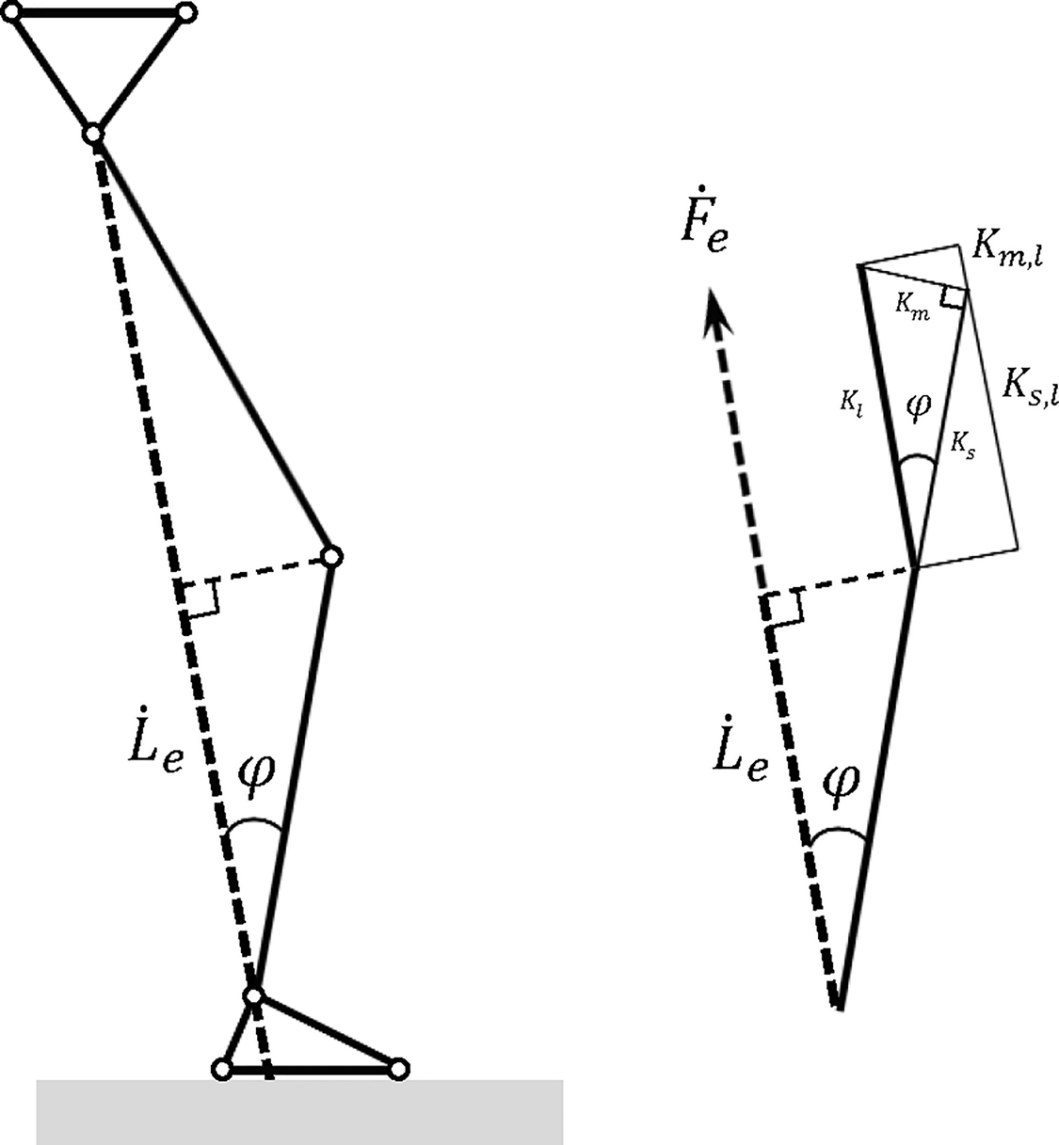
Skeletal and muscular components of the leg stiffness *(K*_*l*_). φ(t) is the angle between the longitudinal axis of the shank and the line joining the COP of the GRF; *K*_*s,l*_ (*t*) is the skeletal component in *K*_*l*_ (*t*); *K*_*m,l*_ (*t*) is the muscular component in *K*_*l*_ (*t*); *K*_*s*_(*t*) is the skeletal stiffness; and *K*_*m*_(*t*) is the muscular stiffness.

To reduce adverse effects from high frequency noise, *F*_*e*_(t), *L*_*e*_(t), (*θ*(t)) and *M*(t) were low-pass filtered using a fourth-order bi-directional Butterworth filter with a cut-off frequency of 10 Hz [19] before being used to calculate the leg and joint stiffness. The obtained leg and joint stiffness were smoothed using a moving average filter with a 50 ms time window [26]. The leg stiffness was further decomposed into skeletal and muscular components as follows [13]:

$$K_{l}\left(t\right)\;=\;K_{m,l}\left(t\right)+K_{s,l}\left(t\right)\;=\;K_{l}\left(t\right){sin}^{2}\varphi \left(t\right)+K_{l}\left(t\right){cos}^{2}\varphi \left(t\right);$$ (7)

$$K_{m,l}\left(t\right)\;=\;K_{m}\left(t\right)sin\varphi \left(t\right);\;K_{s,l}\left(t\right)\;=\;K_{s}\left(t\right)cos\varphi \left(t\right);$$ (8)

$$K_{m}\left(t\right)\;=\;K_{l}\left(t\right)sin\varphi \left(t\right);\;K_{s}\left(t\right)\;=\;K_{l}\left(t\right)cos\varphi \left(t\right);$$ (9)

where φ(t) is the angle between the longitudinal axis of the shank and the line joining the COP of the GRF; *K*_*s*,*l*_ (*t*) is the skeletal component in *K*_*l*_ (*t*); *K*_*m*,*l*_ (*t*) is the muscular component in *K*_*l*_ (*t*); *K*_*s*_(*t*) is the skeletal stiffness; and *K*_*m*_(*t*) is the muscular stiffness (Fig 2). Based on a simplified leg model, Eqs (7–9) have been shown to describe the skeletal and muscular components of the leg stiffness (Fig 2) [13]. The foot segment was not included in the model because the effects of the ankle position on φ(t) was minimal. Since the axial force transmitted by a bone passes through its proximal and distal joints, the skeletal component of the leg stiffness is related to the alignment between the bones, i.e., joint angles, instead of the shape (or deformity) of the bones. A completely extended (φ(*t*) = 0) lower limb would have the leg stiffness provided solely by the skeletal component. As the angle (φ) increases, the contribution of the muscular component to the leg stiffness increases with the increasing lever arm length available to the effective GRF at the knee (Fig 2). The ratio of the muscular and skeletal components was also calculated to assess the relative contribution of the two components (muscular/skeletal).

The leg stiffness, the skeletal and muscular components and their ratios, as well as the angles, moments and stiffness at the hip, knee and ankle in the sagittal plane, were calculated for each of the lower limbs during the gait cycle, both during single limb support (SLS) and double limb support (DLS), using the COP and GRF data measured for each limb. The results were time-averaged over the sub-phases of the stance phase, namely loading response (initial DLS), mid-stance, terminal stance and pre-swing (terminal DLS) for each limb. The data from both limbs were then averaged for each trial for each subject. These gait phases were determined using the forceplate and marker data. Temporal-spatial parameters, namely walking speed, stride length normalized to leg length, stride time, cadence and step width, were also obtained. For data presentation, the curves of each angle and moment component at each joint from all the subjects were ensemble-averaged to obtain the means and standard deviations during the gait cycle for each subject group.

### Statistical analysis

For each of the calculated variables independent t-tests were performed to compare each of the time-averaged values over the sub-phases between the CP and control groups. The effect of gait speed on the between-group comparisons in all stiffness-related variables was tested using analysis of covariance (ANCOVA) with the gait speed as a confounding factor. All significance levels were set at α = 0.05. All the statistical analyses were performed using SPSS version 20.0 (SPSS Inc., Chicago, IL, USA).

## Results

Compared to the healthy controls, the CP group showed significantly increased stride time and step width, but significantly reduced gait speed and cadence (Table 2). However, none of the between-group comparisons in stiffness-related variables were affected by gait speed (p>0.05).

**Table 2:** Means (standard deviations) of the temporal-spatial parameters of gait for children with CP and the controls. Statistical results using an independent-samples t-test are shown. An asterisk indicates a significant group difference (*p* < 0.05).

Group	Cadence (steps/min)	Gait speed (m/s)	Stride time (s)	Stride length (m)	Step width (m)
Control	118.81 (11.19)	1.11 (0.21)	1.01 (0.11)	1.10 (0.12)	0.10 (0.03)
CP	101.50 (19.63)	0.87 (0.20)	1.25 (0.23)	1.04 (0.12)	0.15 (0.08)
p-value	0.014*	0.011*	0.003*	0.216	0.018*

**Fig 3:**
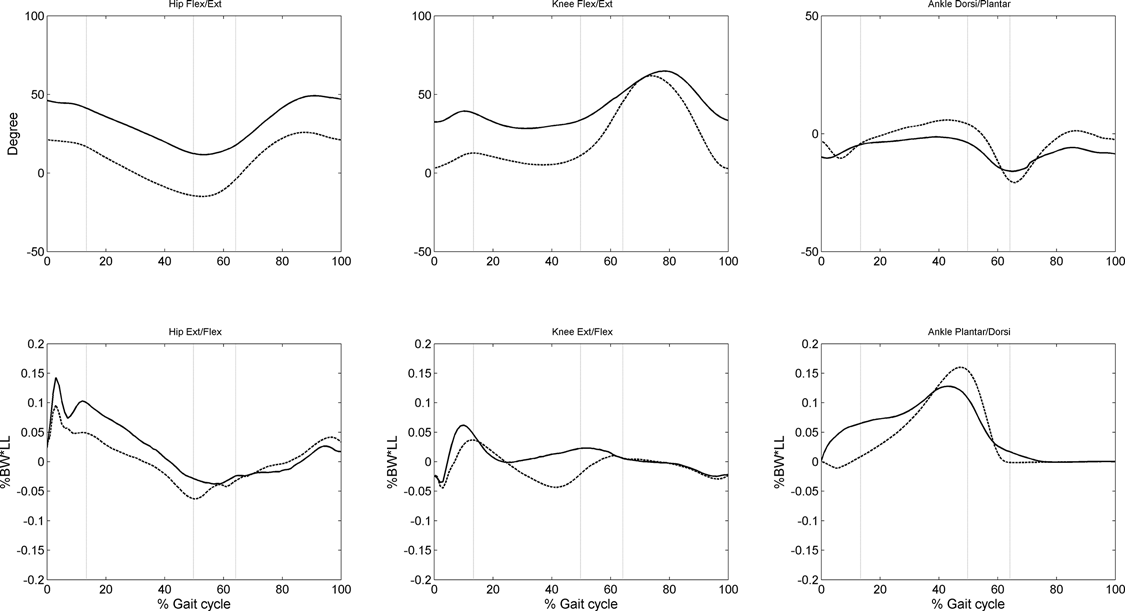
Ensemble-averaged joint angles and moments in the sagittal plane. The angles and moments at the hip, knee and ankle for the control group are shown as dashed lines and those for the CP group as solid lines. %BW*LL indicates the moments normalized to the body weight and leg length.

Compared to the control group, the CP group showed significantly increased flexion at the hip and knee during most of the stance phase, and increased ankle plantarflexion during terminal stance and the pre-swing phase (Fig 3 and Table 3). The CP group also showed significantly increased hip extensor moments during the entire stance phase, and increased ankle plantarflexor moments during loading response and mid-stance, but decreased knee extensor moments during mid-stance, and decreased plantarflexor moments during pre-swing (Fig 3 and Table 3).

**Table 3:** Means (standard deviations) of the time-averaged angles and moments of the hip, knee and ankle in the sagittal plane over each of the sub-phases of the stance phase of gait for children with CP and the control group. An asterisk indicates a significant group difference (*p* < 0.05). %BW*LL indicated the moments normalized to the body weight and leg length.

	Load Response	Mid-Stance	Terminal Stance	Pre-Swing
Hip Angle (degree)				
Control	23.19 (6.08)	12.71 (6.34)	-3.97 (6.14)	-9.03 (5.82)
CP	44.72 (13.21)	35.89 (14.11)	19.80 (15.44)	12.40 (14.57)
p-value	0.000*	0.000*	0.000*	0.000*
Knee Angle (degree)				
Control	12.63 (9.22)	15.95 (8.48)	12.92 (7.89)	27.35 (8.18)
CP	35.57 (9.38)	33.27 (8.20)	30.19 (11.03)	39.30 (10.32)
p-value	0.000*	0.000*	0.000*	0.005*
Ankle Angle (degree)				
Control	-4.12 (4.54)	1.45 (4.77)	6.60 (3.78)	-1.62 (3.11)
CP	-6.89 (7.90)	-3.77 (10.27)	-2.06 (10.35)	-8.64 (10.67)
p-value	0.308	0.125	0.016*	0.048*
Hip moment (%BW*LL)				
Control	0.05 (0.01)	0.02 (0.01)	-0.03 (0.01)	-0.05 (0.01)
CP	0.09 (0.04)	0.08 (0.04)	-0.00 (0.02)	-0.03 (0.02)
p-value	0.007*	0.000*	0.000*	0.015*
Knee moment (%BW*LL)				
Control	0.02 (0.02)	0.05 (0.02)	0.00 (0.00)	0.02 (0.01)
CP	0.01 (0.03)	0.02 (0.03)	0.01 (0.04)	0.02 (0.03)
p-value	0.247	0.005*	0.436	0.701
Ankle moment (%BW*LL)				
Control	- 0.01 (0.00)	0.03 (0.02)	0.13 (0.01)	0.08 (0.02)
CP	0.02 (0.03)	0.07 (0.03)	0.11 (0.03)	0.06 (0.02)
p-value	0.001*	0.000*	0.168	0.044*

Compared with the healthy controls, the CP group showed significantly reduced leg stiffness during loading response and mid-stance, with increased joint stiffness at the hip, knee and ankle (Table 4). During terminal stance no significant differences in leg stiffness were found between the CP and control groups, except for significantly increased joint stiffness at the knee (Table 4). During pre-swing phase, the CP group significantly reduced their leg stiffness with increased joint stiffness at the hip and knee (Table 4).

**Table 4:** Means (standard deviations) of the leg and joint stiffness, and skeletal and muscular components of leg stiffness in children with CP and the control group during each sub-phase of stance during gait. An asterisk indicates a significant group difference (p < 0.05). %BW*LL/degree indicated the moments normalized to the body weight and leg length, and divided by joint angle.

	Load Response	Mid-Stance	Terminal Stance	Pre-Swing
Leg Stiffness (N/m)				
Control	15.02 (6.42)	14.64 (6.27)	9.36 (2.72)	5.52 (2.69)
CP	8.41 (5.22)	9.79 (3.16)	10.17 (3.62)	2.94 (1.95)
p-value	0.011*	0.033*	0.538	0.018*
Skeletal Component (N/m)				
Control	14.69 (6.37)	15.21 (7.43)	8.52 (2.57)	4.03 (2.23)
CP	7.47 (5.12)	9.64 (4.16)	8.82 (3.35)	2.61 (1.77)
p-value	0.006*	0.034*	0.805	0.099
Muscular Component (N/m)				
Control	0.33 (0.18)	0.73 (0.28)	0.84 (0.21)	1.14 (0.65)
CP	0.72 (0.50)	1.01 (0.36)	1.22 (0.61)	0.60 (0.45)
p-value	0.019*	0.045*	0.057	0.032*
Ratio of Muscular & Skeletal components				
Control	2.25%	4.80%	9.86%	28.29%
CP	9.64%	10.48%	13.83%	22.99%
p-value	0.003*	0.025*	0.553	0.429
Hip joint stiffness (%BW*LL /degree)				
Control	0.03 (0.01)	0.003 (0.0006)	0.005 (0.002)	0.006 (0.002)
CP	0.07 (0.05)	0.005 (0.002)	0.006 (0.003)	0.009 (0.004)
p-value	0.013*	0.013*	0.565	0.022*
Knee joint stiffness (%BW*LL /degree)				
Control	0.01 (0.006)	0.012 (0.003)	0.004 (0.001)	0.001 (0.0003)
CP	0.02 (0.007)	0.015 (0.003)	0.01 (0.002)	0.003 (0.002)
p-value	0.032*	0.009*	0.008*	0.003*
Ankle joint stiffness (%BW*LL /degree)				
Control	0.005 (0.001)	0.008 (0.003)	0.02 (0.01)	0.0074 (0.002)
CP	0.007 (0.003)	0.01 (0.01)	0.01 (0.004)	0.0073 (0.003)
p-value	0.020*	0.002*	0.143	0.973

Compared with the healthy controls, the children with CP walked with a significantly increased muscular component but reduced skeletal component of the leg stiffness during loading response and mid-stance, giving a significantly increased ratio of the muscular to skeletal components (Table 4). The muscular component was significantly reduced during the pre-swing phase (Table 4).

## Discussion

The current study was the first attempt to quantify the leg stiffness and the associated skeletal and muscular components, as well as joint stiffness, during stance phase of gait in children with spastic diplegia CP. In the literature, some studies calculated the leg stiffness as the ratio between the peak GRF and maximum displacement [8, 27], giving only a single value to indicate the stiffness. Others calculated a single stiffness value as the slope of the force-displacement curve during the stance phase, approximated using linear regression [28]. Therefore, leg stiffness during the sub-phases was not considered in either approach. In the current study, these approaches were extended via an appropriate division of the stance phase into sub-phases, which enabled a better description of the non-linear leg and joint stiffness changes in the CP gait.

During loading response or initial DLS, children with CP showed a reduced leg stiffness even though the stiffness of individual joints was increased compared to the control group. This appeared to be a result of the more flexed posture of the lower limbs (Fig 3) while accepting the body weight [28], which also increased the share of the muscles in providing the observed leg stiffness as indicated by the increased muscular component (Table 4). The results suggest an increase in difficulty in providing the necessary stiffness of the body weight-accepting leg for controlling the balance of the body, especially for those with muscle weakness. Note that during this period, the contralateral limb was in the pre-swing (or terminal DLS) phase, during which the main task of the stance limb is to transfer the body weight to the weight-accepting ipsilateral limb and prepare for swing. Well-coordinated transfer of the body weight and modulation of the leg and joint stiffness between the two limbs during this period is critical for gait stability in children with CP.

During the pre-swing phase, the reduced leg stiffness but increased joint stiffness in the CP group suggests that the subjects may have a compromised ability for providing a pushing-off force for the subsequent swing. Since the body weight is transferring from the stance limb to the contralateral limb, the leg stiffness and the walking stability during this period did not have to be as high as that during weight acceptance. However, instability in the control of the stance limb during the unloading phase may affect the smooth transfer of the body weight, leading to an increased joint stiffness and muscular component of leg stiffness. The reduced leg stiffness but increased joint stiffness in both limbs during DLS suggest that the children with diplegic CP had altered coordination of the transfer of the body weight and modulation of the leg and joint stiffness between the limbs, leading to a higher risk of imbalance or collapse during weight transfer.

During mid-stance, the objective was to ensure smooth progression of the body over the stationary foot, which had to be based on necessary stability of the lower limb. Following loading response, the more flexed posture of the lower limbs during this period also contributed to the reduced leg stiffness in the children with CP, requiring increased muscular components and joint stiffness. This suggests that when subjected to a sudden increase in the GRF, e.g., extra force needed to recover balance, the risk of collapse in these children may increase.

During terminal stance, the CP group increased the leg stiffness primarily via increased knee joint stiffness, presumably to prepare for transferring the body weight to the contralateral limb. This would also be helpful for preventing an undesired reduction in toe clearance of the contralateral limb owing to increased shortening of the ipsilateral leg length. This is particularly important in preventing stumbling as their ankle dorsiflexion angles were reduced (Fig 3). It appears that increasing the leg stiffness helps prevent the knee joint from becoming overflexed by the GRF, contributing to a larger stride length of the swing limb and smoother body progression.

The CP group appeared to rely more on muscular contributions to achieve the required leg stiffness. The more flexed hip and knee increased the lever-arm length of the GRF vector at the joint centers, and thus the joint moments. This also increased the contribution of the musculature system to the leg stiffness but decreased that of the skeletal system [29]. The increased muscular component placed a further challenge on the joints given their impairments, including reduced strength [4].

With a more flexed posture during the stance phase, the CP group had to sustain increased loads at the individual joints, as well as increased joint stiffness. However, these changes did not maintain the leg stiffness within the normal range. This appeared to be related to the inability to modulate the leg stiffness to compensate for the symptoms of the neurological changes and the muscle weakness during gait (Table 1). Decreased leg stiffness during weight acceptance in the children with CP induced an increase in their muscular demands for maintaining the body posture against collapse. On the other hand, increased demands on the extensor muscles during weight unloading are also essential for the support and progression of the body with the contralateral limb. Other factors such as muscle spasticity, contracture and joint deformity would also affect the modulation and control of the joint and leg stiffness. Since the current patients showed only mild spasticity and slight contracture at the ankle with slightly limited ROM, but without obvious deformity (Table 1), muscle strength and selective control appeared to be a main factor. Therefore, strengthening and stretching of the lower limb muscles is considered essential for improving the control of the leg stiffness in order to maintain functionality of the overall lower extremity [30]. Nonetheless, further studies will be needed to better establish the roles of specific factors in the control of leg and joint stiffness in children with different types of CP.

The leg stiffness and related variables proposed in the current study provide a measure for assessing the control of the lower extremities as a whole in supporting the body against collapse during gait, which would be very helpful in the assessment of the effects of the pathology and the efficacy of relevant treatment methods in a clinical setting. This information is not readily available from the individual joint angular and moment data, nor from the EMG data of the relevant muscles obtained using traditional gait analysis. It is thus suggested that the stiffness-related variables should be included in future clinical gait analysis for a more complete assessment of gait in children with CP. As a first step, the current study demonstrated that these variables successfully distinguish the control of the lower limb as a whole between diplegic CP and healthy subjects, providing baseline data for these subject groups. On the other hand, while leg stiffness-related variables integrate both force and displacement information (Eq 7), how these stiffness data, the muscular component in particular, can be related to muscle EMG requires further investigation. Once the interrelationship between the traditional gait variables and the proposed stiffness variables is established either qualitatively or quantitatively, a more complete assessment of pathological gait and its treatment can be achieved. For the use of the proposed stiffness-related variables in therapeutic decision-making and planning in children with CP, however, further study is needed to determine the effects of specific therapeutic treatments, such as Botox injection, exercise training, casts or surgery, in terms of these variables. Besides, the current study was limited to diplegic cerebral palsy. Further study is also needed for determining any differences in the biomechanical strategies adopted by different types and severities of the disease in order to develop better training programs.

## Conclusions

Leg stiffness plays a critical role in modulating the kinematics and kinetics of the locomotor system during gait in diplegic CP. The CP group significantly decreased the leg stiffness but increased the joint stiffness during stance phase, except during terminal stance where the leg stiffness was increased. They appeared to rely more on muscular contributions to achieve the required leg stiffness, increasing the muscular demands in maintaining the body posture against collapse. Therefore, therapeutic decision-making and planning should consider not only the mechanics of individual joints, but also the leg stiffness control. The stiffness-related variables should be included in future clinical gait analysis for a more complete assessment of gait in children with CP.

## Supporting Information

S1 Dataset Data contains the stiffness-related variables of CP and the controls during four phases of gait cycle. (CSV)
